# Uracil DNA N-Glycosylase Promotes Assembly of Human Centromere Protein A

**DOI:** 10.1371/journal.pone.0017151

**Published:** 2011-03-02

**Authors:** Samantha G. Zeitlin, Brian R. Chapados, Norman M. Baker, Caroline Tai, Geir Slupphaug, Jean Y. J. Wang

**Affiliations:** 1 Moores UCSD Cancer Center and Ludwig Institute for Cancer Research, University of California San Diego, La Jolla, California, United States of America; 2 Department of Molecular Biology, The Scripps Research Institute, La Jolla, California, United States of America; 3 Department of Bioengineering, University of California San Diego, La Jolla, California, United States of America; 4 Moores UCSD Cancer Center, University of California San Diego, La Jolla, California, United States of America; 5 Faculty of Medicine, Norwegian University of Science and Technology (NTNU), Trondheim, Norway; Texas A&M University, United States of America

## Abstract

Uracil is removed from DNA by the conserved enzyme Uracil DNA N-glycosylase (UNG). Previously, we observed that inhibiting UNG in Xenopus egg extracts blocked assembly of CENP-A, a histone H3 variant. CENP-A is an essential protein in all species, since it is required for chromosome segregation during mitosis. Thus, the implication of UNG in CENP-A assembly implies that UNG would also be essential, but UNG mutants lacking catalytic activity are viable in all species. In this paper, we present evidence that UNG2 colocalizes with CENP-A and H2AX phosphorylation at centromeres in normally cycling cells. Reduction of UNG2 in human cells blocks CENP-A assembly, and results in reduced cell proliferation, associated with increased frequencies of mitotic abnormalities and rapid cell death. Overexpression of UNG2 induces high levels of CENP-A assembly in human cells. Using a multiphoton laser approach, we demonstrate that UNG2 is rapidly recruited to sites of DNA damage. Taken together, our data are consistent with a model in which the N-terminus of UNG2 interacts with the active site of the enzyme and with chromatin.

## Introduction

Deoxyuridine is both generated in DNA by cytidine deamination and incorporated during DNA replication (reviewed in [1]). Uracil is rapidly removed from DNA by the highly conserved enzyme Uracil DNA N-glycosylase (UNG), which has two isoforms in mammalian cells, UNG1 (mitochondrial) and UNG2 (nuclear) [2]. High levels of uracil in DNA result when folate levels are low, e.g due to dietary deficiency or prolonged methotrexate treatment, or when dUTPase is inhibited [3]. High levels of uracil in DNA causes chromosome breakage and increased rates of mutation, leading to increased cancer rates (reviewed in [4]).

Previously, in vitro evidence identified a role for UNG2 upstream of CENP-A assembly both at centromeres and other sites of DNA damage, since inhibiting UNG2 completely block formation of any CENP-A foci [5]. CENP-A is an essential histone H3 variant, required to mediate kinetochore assembly for chromosome segregation during mitosis (reviewed in [6]). CENP-A also binds to sites of DNA damage and appears to have a role in DNA repair [7]. It is currently somewhat unclear whether CENP-A assembles into canonical nucleosomes at centromeres, and it seems unlikely that CENP-A forms nucleosomes at all sites of DNA damage (reviewed in [6]).

In addition to its known role in removing uracil, a requirement for UNG2 in CENP-A recruitment to DNA or “CENP-A assembly” implies a role for UNG2 at centromeres. However, UNG mutants are viable in all species, from bacteria [8] to yeast [9] to worms [10], while mice lacking the catalytic domain of UNG2 are viable and exhibit relatively minor abnormalities [11]–[13]. Humans and mice with mutations in UNG2 exhibit primarily immunological defects, due to failures in somatic hypermutation and class switch recombination [14]–[19].

Despite the relatively limited phenotypes of mammals with UNG2 mutations, other groups have observed that UNG2 reduction resulted in reduced proliferation of human cells [3], [20] and that UNG2 inhibition resulted in mammalian cell death [21], [22]. One way to reconcile these results is if mutations in the active site of UNG2 are not equivalent to loss of protein interactions via reduction of the protein levels or sequestration via inhibitor binding. Alternatively, it has been suggested that UNG2 is only essential after induction of exogenous damage, such as ionizing radiation [23].

It is possible that the N-terminus plays an underappreciated role in UNG2 localization and function. Most of the existing measurements of UNG2 catalytic activity refer to the catalytic domain alone, because the N-terminus is rapidly removed by proteolytic cleavage during purification [24]. For that reason, all of the existing crystal structures of UNG lack the N-terminus (see for example [25]–[28]). A series of reports by another group using full-length purified UNG2 have implicated interactions of the N-terminus with the active site [29]–[31]. This model could potentially resolve the earlier conflicting observations regarding the roles of UNG2 in vivo.

Since the initial implication of UNG2 in CENP-A assembly, many other proteins have been proposed as CENP-A assembly factors in metazoans (reviewed in [6]). Subsequently, we reported that double-strand breaks induced by a laser or a specific endonuclease can rapidly recruit CENP-A and several of its associated kinetochore proteins [7]. Since uracil removal on opposite strands of DNA can result in double-strand breaks [32], [33], the role of double-strand breaks in CENP-A recruitment did not rule out a role for UNG2 at centromeres in normally cycling cells.

In this paper, we present evidence that UNG2 colocalizes with CENP-A and H2AX phosphorylation at centromeres in normally cycling cells. Reduction of UNG2 in human cells blocks CENP-A assembly, consistent with our previous in vitro results from Xenopus extracts [5]. Our results also corroborate reports that UNG2 reduction results in reduced cell proliferation, associated with increased frequencies of mitotic abnormalities and rapid cell death. Using a multiphoton laser approach [7], we demonstrate that UNG2 is rapidly recruited to double-strand breaks in vivo, independent of its catalytic activity. Taken together, our data are best explained by a model in which the N-terminus of UNG2 interacts with the active site of the enzyme and with chromatin.

## Results

### Ectopic expression of the HIV protein Vpr causes CENP-A protein loss

**Figure 1:**
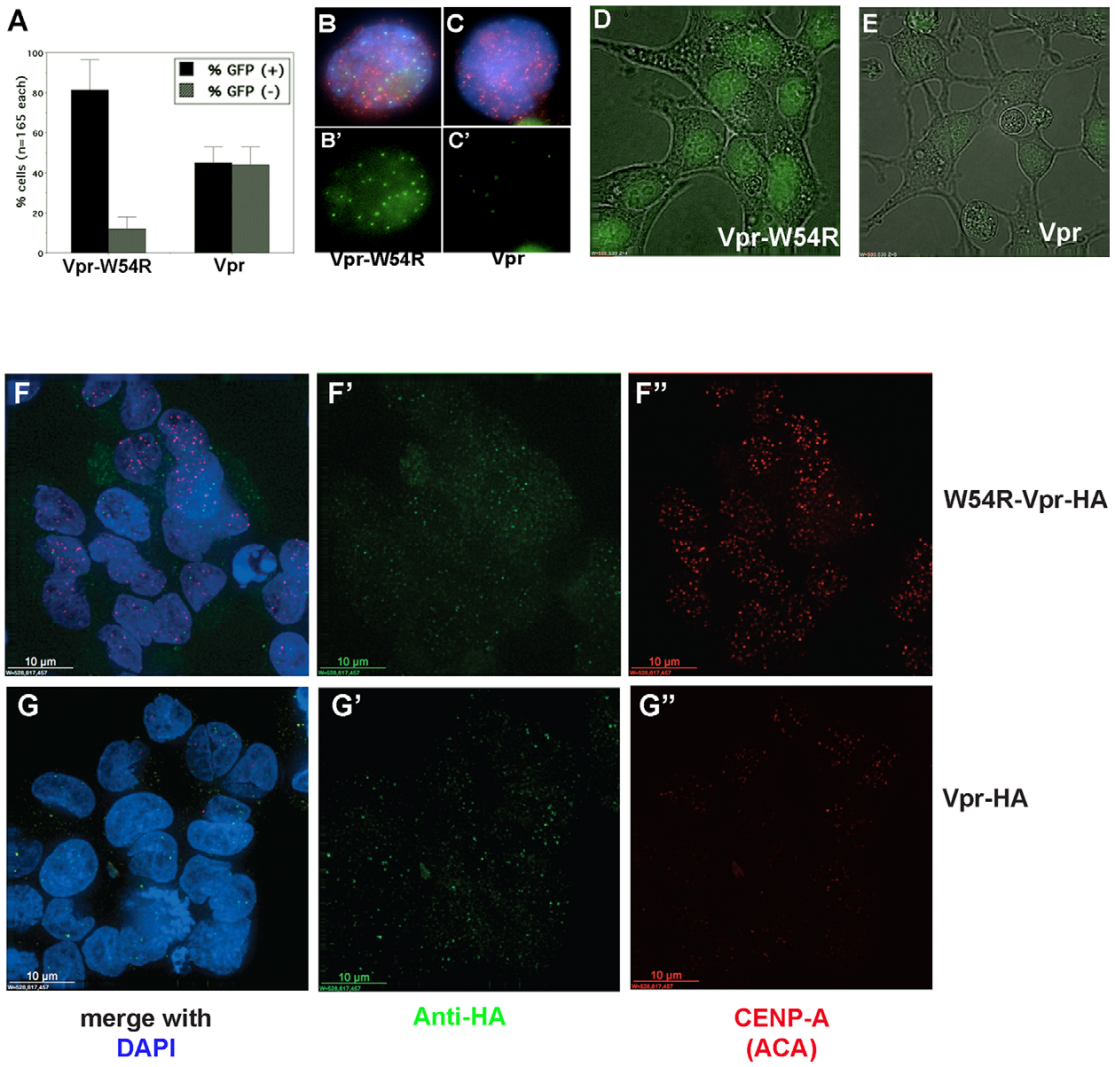
Transient transfection to induce Vpr expression destabilizes both endogenous and GFP-CENP-A. A. Manual quantitation of GFP-CENP-A cells after transfection with control Vpr mutant W54R or wild-type Vpr. B–C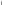. Example images of cells transfected with control Vpr-W54R-HA (B-B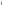) or wild-type Vpr-HA (C-C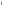) detected with anti-HA in red, DNA in blue, and GFP-CENP-A in green. GFP-CENP-A channel is also shown alone (B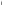 and C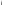). D–E. Example fields of live GFP-CENP-A cells transfected with Vpr-W54R (D) or wild-type Vpr (E). F–G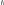. Example fields of fixed HeLa cells after transfection with Vpr-W54R (F-F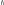) or wild-type Vpr (G-G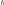). Vpr is detected with anti-HA in green. Endogenous CENP-A is detected with anti-centromere autoantisera (ACA) in red.

Previously, we observed that inhibiting UNG2 in Xenopus egg extracts was sufficient to block assembly of Xenopus CENP-A [5]. Based on the finding that wild-type Vpr induces a loss of UNG2 when transiently transfected into HeLa cells, but the W54R mutant of Vpr does not [34], we used this as an approach to testing the effect of reduced UNG2 levels on CENP-A in human cells. Wild-type or the W54R mutant Vpr were tagged with HA and transiently transfected into tet-inducible GFP-CENP-A Hek293 cells [7]. GFP-CENP-A was induced by addition of tetracycline, and the Vpr constructs were detected by indirect immunofluorescence in fixed cells using a monoclonal antibody against the HA tag. Cells displaying signal for Vpr (WT or W54R, detected in red) were scored for the presence or absence of GFP-CENP-A signal (Figure 1A, example images are shown in B-CÕ). This analysis demonstrated that a significant fraction of cells expressing wild-type Vpr had little or no detectable GFP-CENP-A.

To determine whether the GFP-CENP-A was mislocalized or being extracted during fixation, a similar experiment was performed to observe the GFP-CENP-A signal in live cells using confocal microscopy (Figure 1, D–E). This experiment demonstrated that GFP-CENP-A is visibly reduced in cells transfected with Vpr. Similar results were seen using immunofluorescence to detect endogenous CENP-A in HeLa cells transfected with wild-type or W54R Vpr, detected with HA (Figure 1 F–G). Taken together, these data indicate that Hek293 and HeLa cells transiently transfected with the HIV protein Vpr exhibit lower levels of CENP-A protein.

### UNG2-directed siRNA causes GFP-CENP-A loss

**Figure 2:**
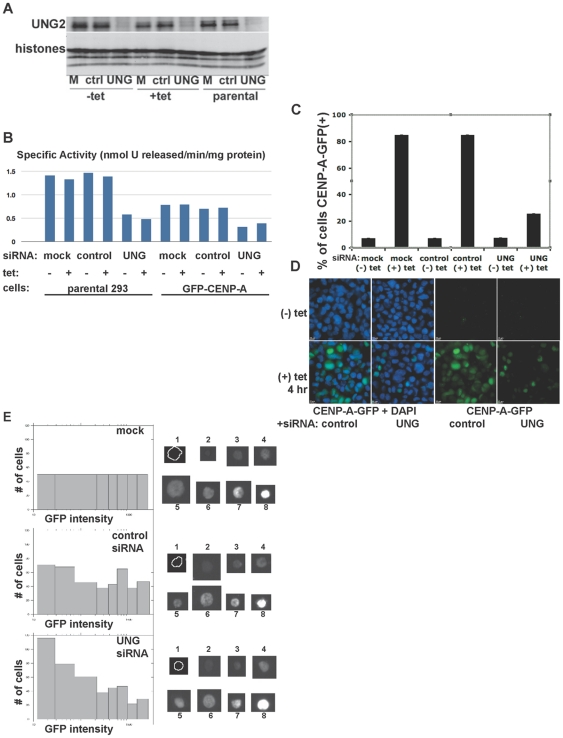
Transient transfection of UNG-directed siRNA destabilizes both endogenous UNG2 and GFP-CENP-A. A. Western blot analysis of UNG2 protein levels (top) and endogenous histones as a loading control (bottom) in parental Hek293 cells, uninduced or induced to express GFP-CENP-A. B. Specific activity of cellular extracts for uracil removal with and without transfection of control or UNG-directed siRNA. Parental Hek293 cells, uninduced (-tet) cells and mock transfected cells were used as controls. C. Manual quantitation of GFP-CENP-A cells with and without transfection of UNG-directed siRNA. Uninduced (-tet) cells were used as controls. D. Example images of cells used for quantitation shown in part C. DNA was detected with DAPI (blue) to identify nuclei. E. High-throughput (n = 5000 cells per well, 3 wells per sample) automated quantitation (left) and example images (right) using samples generated by the same methods used in parts C and D.

Since the mechanism(s) of how Vpr induces UNG2 loss are controversial [35], [36], we next applied transient transfection of double-stranded siRNA oligonucleotides as an alternative and potentially more specific method for reducing UNG2. First, we verified that UNG2 protein levels were reduced in the parental 293 cells, as well as the GFP-CENP-A expressing cells with and without tetracycline (Figure 2A). Next, we verified that the specific activity of cell lysates in a uracil-removal assay was reduced in samples transfected with UNG2-directed siRNA, but not in mock-transfected or cells transfected with a non-specific control siRNA (Figure 2B; Table 1). This experiment also verified that similar results were seen with and without addition of tetracycline in both parental 293 and stable GFP-CENP-A cells. Although the starting levels of uracil-removing activity were higher in parental 293 cells than in the GFP-CENP-A cells, the overall reduction in activity was similar after siRNA transfection (about 50%). These observations demonstrate that this siRNA approach reduces both UNG2 protein and activity under the conditions used here.

**Table 1:** UNG activity levels in tet-inducible GFP-CENP-A Hek293 cell lysates after siRNA treatment.

Sample	Specific Activity (nmol U/min/mg protein)
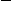 tet, mock	0.78
+tet, mock	0.79
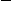 tet, control siRNA	0.70
+tet, control siRNA	0.72
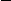 tet, UNG siRNA	0.31
+tet, UNG siRNA	0.39

Next, we tested the effect of UNG2 reduction by siRNA on GFP-CENP-A expression. Cells were mock-transfected, or transfected with control nonspecific or UNG-directed siRNA for 20 hours. Then, GFP-CENP-A expression was induced by addition of tetracycline and cells were further incubated for 4 more hours. Cells were scored for the presence or absence of GFP-CENP-A signal either manually by the hundreds (Figure 2C, examples are shown in D) or using automated image collection and analysis (Cellomics) by the tens of thousands (n = 5000 cells per well, each condition in triplicate wells, Figure 2E). The frequency of cells with visible GFP-CENP-A levels was dramatically reduced in cells transfected with UNG2-directed siRNA, but not with control nonspecific siRNA, relative to untransfected controls (Figure 2C and D). In addition, the overall intensity distribution of the GFP-CENP-A signal was shifted down (Figure 2E). These results are statistically significant (Table 2), and demonstrate that UNG2 reduction by siRNA produces a similar effect on GFP-CENP-A to that observed with Vpr. Taken together, these findings demonstrate that GFP-CENP-A is reduced when UNG2 is depleted by Vpr or siRNA.

**Table 2:** Significance analysis of GFP-CENP-A intensity histogram binning shown in Figure 2E.

Sample	T(X)	p 1-tailed	p 2-tailed
mock vs. control siRNA	0.94	0.17	0.35
control siRNA vs. UNG siRNA	4.44	4.46×10 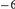	8.92×10 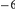
mock vs. UNG siRNA	11.85	1.10×10 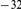	2.20×10 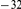

### GFP-CENP-A loss after UNG2 reduction is not due to not cell-cycle arrest or transcriptional effects

**Figure 3:**
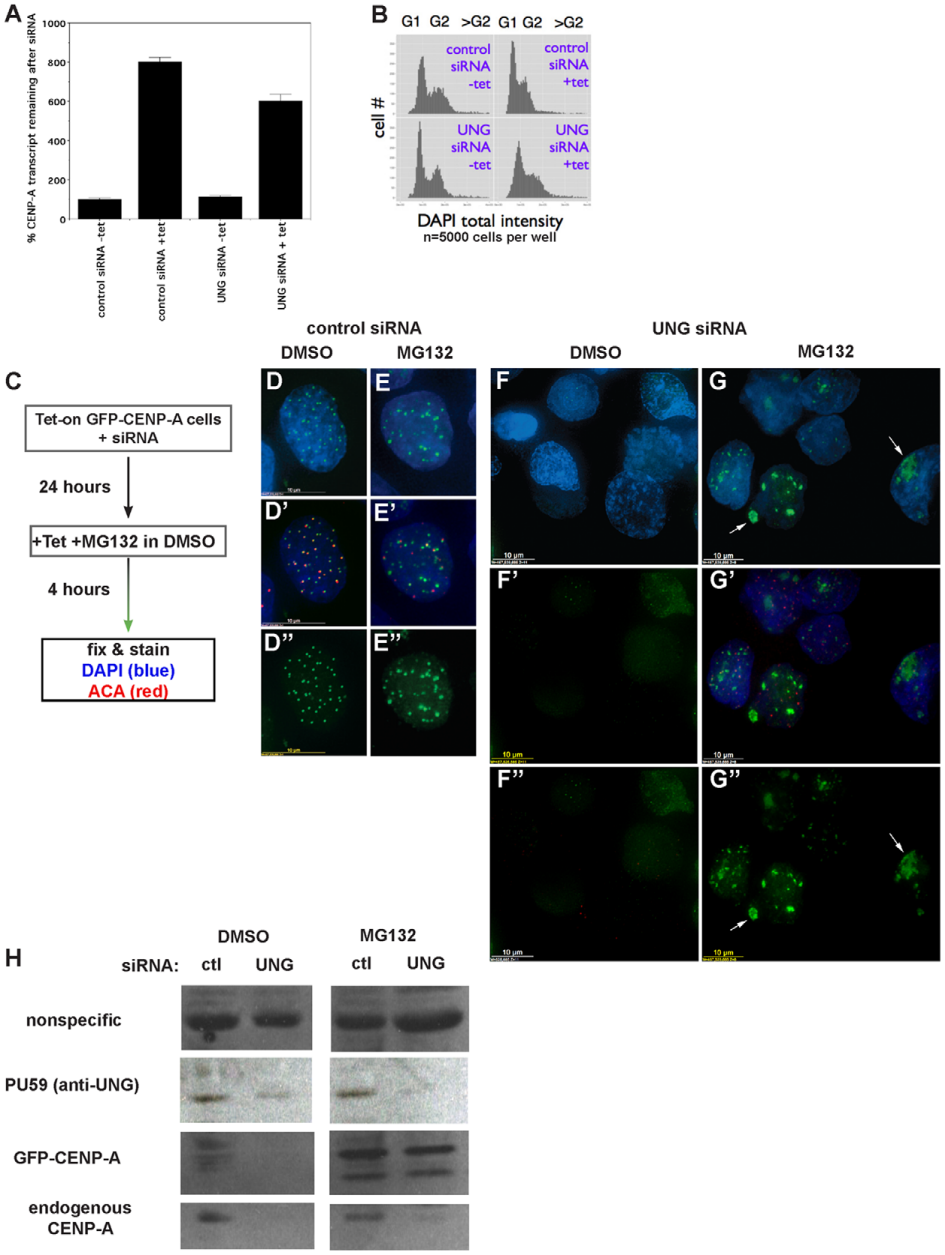
Loss of newly-made GFP-CENP-A after UNG-directed siRNA treatment is due to mislocalization and destabilization, not cell-cycle arrest or lack of synthesis. A. Quantitative real-time RT-PCR was used to detect levels of CENP-A transcript (endogenous and GFP-CENP-A combined) with and without UNG-directed siRNA or tetracycline. Percent CENP-A transcript levels were normalized against the sample with control siRNA without tetracycline. B. Cell cycle analysis was performed using high-content automated image quantitation, with and without UNG-directed siRNA or tetracycline. C–G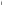. GFP-CENP-A cells were transfected with UNG-directed siRNA were treated with MG132 for 4 hours to prevent protein turnover. A scheme is shown in part C, example DMSO-treated control cell (D-D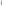), example MG132-treated control cell (E-E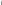). Example fields are shown for UNG-siRNA transfection with DMSO (F-F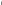) or MG132 (G-G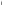). Endogenous CENP-A was detected with human autoantisera (ACA) in red. H. Western blot analysis of protein levels demonstrates that GFP-CENP-A is selectively stabilized by MG132 treatment after UNG reduction by siRNA, while endogenous CENP-A protein levels remain low.

Since the reported effects of Vpr include transcriptional effects [35] and cell-cycle arrest (reviewed by [37], [38], we explored whether the UNG2-directed siRNA might also induce these phenotypes. First, we determined that UNG2-directed siRNA does not inhibit CENP-A transcription, by detecting mRNA using quantitative RT-PCR with primers that detect both endogenous and GFP-CENP-A transcripts (Figure 3A). Next, we determined that UNG2-directed siRNA does not induce a significant cell-cycle arrest when freely cycling cells are treated for 24 hours (Figure 3B). These experiments demonstrate that the UNG2-directed siRNA exhibits a more specific effect than those reported for Vpr.

### Loss of CENP-A after UNG2 reduction is due to mislocalization and degradation

To test whether the absence of GFP-CENP-A in the presence of UNG2-directed siRNA might be due to a lack of GFP-CENP-A protein synthesis, decreased GFP-CENP-A protein stability, or mislocalization of the GFP-CENP-A protein, we induced GFP-CENP-A expression in the presence or absence of the proteasome inhibitor MG132. Cells were transfected with control or UNG2-directed siRNA for 24 hours, and tetracycline was added in the presence of MG132 or solvent alone (DMSO) for 4 hours (Scheme, Figure 3C). In cells transfected with control siRNA, GFP-CENP-A was detected as nuclear foci colocalizing with endogenous CENP-A (detected with ACA, red) with DMSO or MG132 (Figure 3D and E), consistent with centromeres or sites of DNA damage as seen previously [7]. In cells transfected with UNG2-directed siRNA, GFP-CENP-A and endogenous CENP-A were not detectable in the presence of DMSO (Figure 3F), consistent with our initial observations (see Figure 2, C–D). In cells transfected with UNG2-directed siRNA, GFP-CENP-A signal was visible as large aggregates after treatment with MG132, but mislocalized and sometimes cytoplasmic (Figure 3G, arrows). Some endogenous CENP-A foci were detectable in samples treated in MG132 (Figure 3G 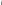, red). These observations suggested that CENP-A might be degraded after UNG2 reduction.

To test whether CENP-A protein was being quantitatively degraded, western blot analysis was performed. This approach demonstrated that GFP-CENP-A protein was detected as leaky expression even in uninduced samples transfected with control siRNA, but this low level was not detected in parallel samples transfected with UNG-directed siRNA. Despite the slight decrease in uninduced samples, the steady-state protein levels were not visibly lowered in samples induced to overexpress GFP-CENP-A. These results indicate that although GFP-CENP-A is mislocalized, and MG132 appears to induce aggregation and possibly accumulation of mislocalized protein, the GFP-tagged CENP-A protein itself is not completely degraded (Figure 3H). In contrast, this experiment revealed that endogenous CENP-A protein levels decreased dramatically after UNG2 reduction. This observation suggests that the N-terminal GFP tag may protect CENP-A from proteolytic degradation when UNG2 is absent. Taken together, these results demonstrate that when UNG2 levels are reduced, GFP-CENP-A is synthesized, but it is somewhat degraded and largely mislocalized. In contrast, endogenous CENP-A is no longer detectable after UNG2 reduction.

### UNG2-directed siRNA causes endogenous CENP-A loss and mitotic delay

**Figure 4:**
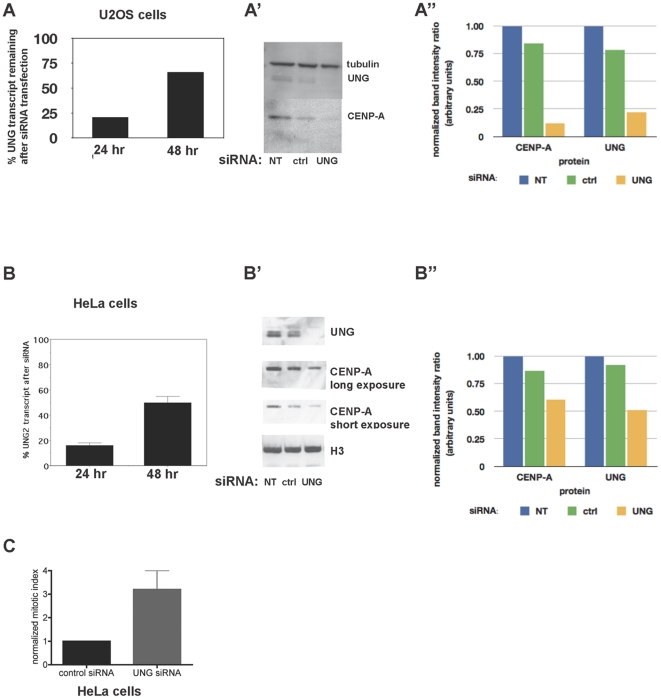
UNG-directed siRNA destabilizes endogenous CENP-A in U20S and HeLa cells. A. Quantitative real-time RT-PCR was used to detect levels of UNG transcript remaining after siRNA transfection in U20S cells. A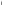. Western blot analysis of endogenous UNG2 and CENP-A protein levels 48 hours after siRNA transfection in U20S cells. Tubulin was detected as a loading control. A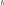. Normalized band intensity for part A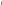. B. Quantitative real-time RT-PCR was used to detect levels of UNG transcript remaining after siRNA transfection in HeLa cells. B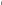. Western blot analysis of endogenous UNG2 and CENP-A protein levels 24 hours after siRNA transfection in HeLa cells. Histone H3 was detected as a loading control. B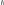. Normalized band intensity ratios for part B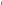. C. Normalized mitotic index of HeLa cells 24 hours after transfection with control or UNG-directed siRNA (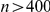 cells per sample). 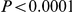 (unpaired t-test).

To test whether endogenous CENP-A protein is consistently lost after UNG2 reduction, two experiments were performed. First, two additional cell lines were subjected to reduction of UNG2 via siRNA treatment. U2OS cells displayed a reduction in UNG2 transcript detected by quantitative RT-PCR (Figure 4A, top) and protein detected by western analysis (Figure 4A, bottom). These cells also displayed a reduction in CENP-A protein, after transfection of UNG2-directed siRNA, similar to that seen in Hek293 cells. HeLa cells behaved similarly, in that they also displayed a reduction in UNG2 transcript detected by quantiative RT-PCR (Figure 4B, top) and protein detected by western analysis, this time using a different CENP-A antibody (Figure 4B, bottom). Taken together with Figure 3H, these experiments demonstrate the failure to detect endogenous CENP-A protein after UNG2 reduction in three different cell lines from different cell types, i.e. human embryonic kidney, osteosarcoma, and cervical carcinoma.

Next, because reduction or loss of CENP-A is known to induce mitotic delays and defects (reviewed in [6]), the mitotic figures of HeLa cells were examined 24 hours after transfection with control or UNG2-directed siRNA. HeLa cells were chosen for these experiments because these cells express higher levels of CENP-A, the observation of intermediate phenotypes requires an intermediate level of protein reduction. This analysis revealed a more than 3-fold increase in mitotic index in cells transfected with UNG2-directed siRNA. This suggested that a partial reduction of UNG2 was sufficient to induce a delay in mitosis, consistent with the observed reduction in CENP-A levels.

### Pleiotropic effects of UNG-directed siRNA on cell division

**Figure 5:**
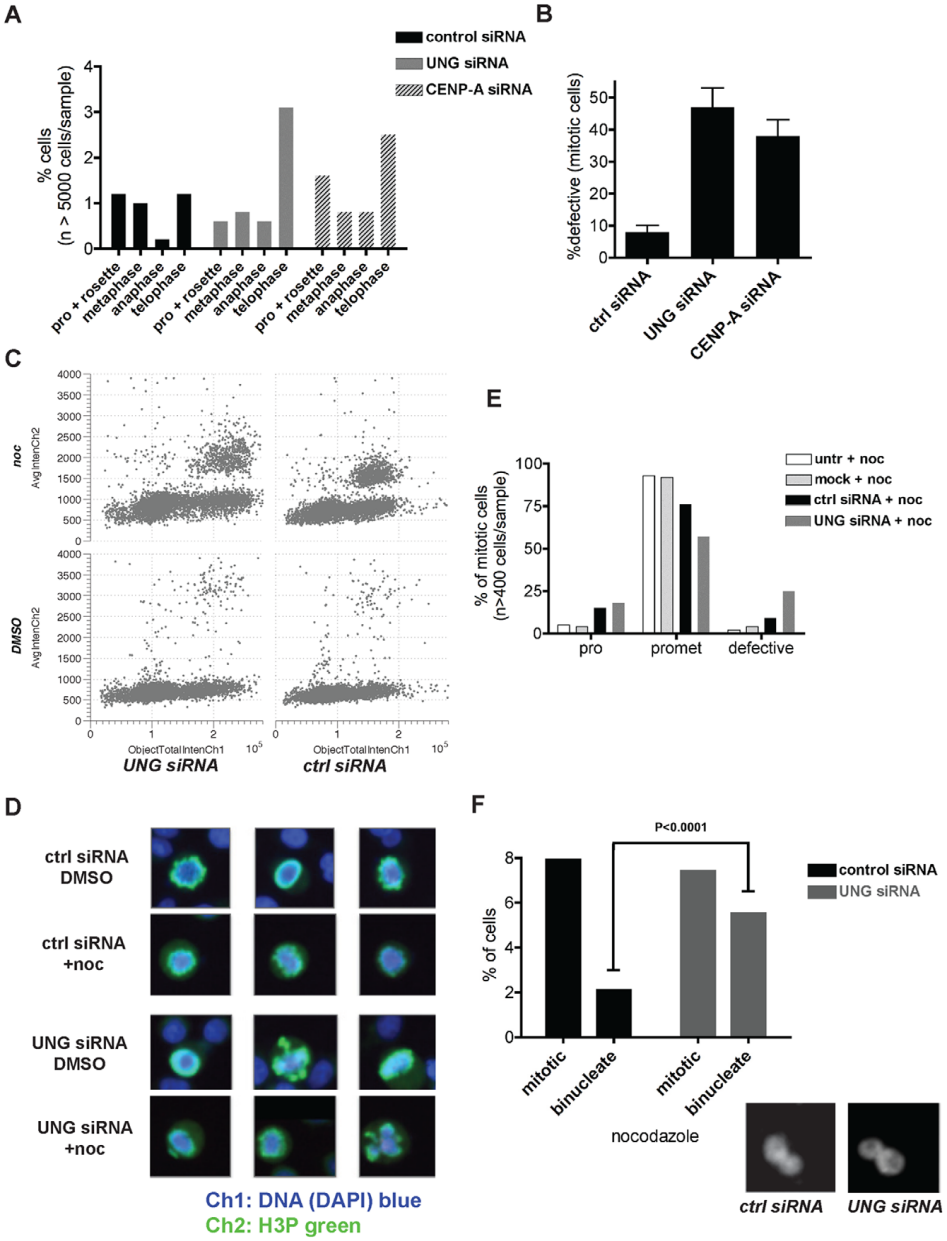
Pleiotropic effects of UNG-directed siRNA on cell division. A. Manual quantitation of H2B-YFP HeLa cells in each stage of mitosis 24 hours after transfection with control siRNA (black bars), UNG-directed (gray bars) siRNA, or CENP-A-directed siRNA (striped bars). B. Relative quantitation of defective mitotic HeLa cells after transfection with control siRNA, UNG-directed siRNA, or CENP-A-directed siRNA. C. High-throughput (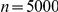 cells per sample) cell cycle analysis was performed using automated quantitative imaging with DAPI to detect DNA content (Total Intensity Ch1, x-axis) and H3P to detect G2 and mitotic cells (Average Intensity Ch2, y-axis). HeLa cells were treated with nocodazole to trigger a mitotic spindle checkpoint arrest, or DMSO solvent alone (bottom row), with or without siRNA transfection with UNG-directed siRNA (left column) or control siRNA (right column). D. Example images from analysis shown in parts C, E and F. DNA was detected with DAPI (blue); Histone H3 phosphorylated on Serine 10, H3P (pseudocolored green), was used to detect cells in G2 (not shown) and mitosis. Defective cells were apparent in samples transfected with UNG-directed siRNA (example, third row, middle) and were more frequent in the presence of nocodazole (examples, bottom row, ends). E. Manual quantitation of HeLa cells after nocodazole treatment. Metaphase and anaphase cells were not seen with nocodazole treatment, consistent with the majority of cells arresting in prometaphase. Samples treated with transfection reagent alone (mock) were similar to untreated samples (untr). Samples transfected with UNG siRNA exhibited fewer cells in prometaphase, and 4-fold more defective mitotic figures than did samples transfected with control siRNA. 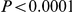 (one-way ANOVA). F. Comparison of cells in mitosis or binucleated after nocodazole treatment, with and without siRNA transfection with control (black bars) or UNG-directed (gray bars) siRNA. 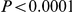 (unpaired t-test).

Detailed analysis of the mitotic populations of freely cycling cells treated with control or UNG-directed siRNA demonstrated modest differences in the fraction of cells detected in the first few stages of mitosis (prophase, rosette/prometaphase Figure 5A). The most striking difference was an apparent 2–3 fold increase in anaphase and telophase cells when compared to cells transfected with control siRNA. Chromosome bridges in anaphase and telophase are associated with DNA repair defects (reviewed in [39]) and kinetochore defects (reviewed in [40]), both of which would be expected when CENP-A levels are reduced. Visual inspection revealed defects in approximately half of the mitotic cells transfected with either UNG siRNA or CENP-A siRNA for 24 hours (Figure 5B), consistent with other reports where CENP-A was depleted using siRNA in human cells (see for example, [41]).

To test whether the apparent accumulation of cells in telophase was a more general phenomenon, and whether the mitotic checkpoint was intact in cells with reduced UNG2 levels, population analysis was performed by automated quantitative imaging (as in Figure 2E). In these experiments, total DNA intensity was measured using DAPI staining (Ch1), and mitotic cells were labeled using H3 phosphorylation (Ch2). Measurements were taken from 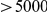 nuclei per sample, and 2D scatterplots were generated for cell cycle analysis [42]. Cells transfected with UNG siRNA rapidly accumulated cells with 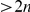 DNA content (DNA content on the x-axis, Figure 5C). While mitotic cells transfected with the control siRNA had normal morphology with or without nocodazole, cells transfected with the UNG siRNA were clearly defective, and even more so in the presence of nocodazole (examples shown in Figure 5D). In some cases, the percentage of mitotic cells with abnormal morphology after UNG siRNA was somewhat lower, e.g. only 25% in the example graph shown in Figure 5E, although this frequency was still 4-fold higher than cells transfected with control siRNA (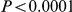, one-way ANOVA).

We wondered what would account for such high variability in the frequency of defective mitoses (compare 45% in Figure 5B with 25% in Figure 5E), and reasoned that cells failing in mitosis should accumulate as binucleates and multinucleates. Cells transfected with UNG2-directed siRNA for only 24 hours consistently exhibited an almost three-fold increase in binucleated cells (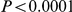, unpaired t-test), compared with cells transfected with control nonspecific siRNA (Figure 5F). Taken together, these results demonstrate defects in cell division after UNG2 depletion, consistent with a reduction of CENP-A.

Since mitotic cells account for only a tiny fraction of a freely cycling cell population (mitosis lasts 1 hour out of a 24 hour cell cycle), the best way to examine the mechanics of mitosis without the potential side-effects of inhibitors is to simply observe cells dividing. For this approach, HeLa cell lines with an integrated GFP-tagged histone H2B were transfected with control or UNG2-directed siRNA, and subjected to time-lapse imaging. This analysis revealed obvious delays and defects in mitosis, consistent with those observed in fixed populations (Movies S1, S2, S3, S4). Most striking, however, was the observation that a larger fraction of the cells transfected with UNG2-directed siRNA were dying (Movies S1, S2). Cell tracking confirmed that these cells were not dying simply due to phototoxicity, which tends to affect all cells simultaneously. Instead, these cells were apparently dying at different times in the cell cycle. This result was somewhat unexpected, given that UNG2 is itself cell-cycle regulated, and given the proposal that CENP-A assembly occurs only during G1 (reviewed in [6]). Our own more recent results demonstrate that CENP-A can assemble throughout the cell cycle [7], suggesting that these cells were dying due to lack of CENP-A assembly after depletion of UNG2, with some variability due to the stochastic extent of transfection, RNAi kinetics, and potentially elevated levels of unrepaired spontaneous DNA damage.

### Depleting or inhibiting UNG2 induces cell death with hallmarks of apoptosis

**Figure 6:**
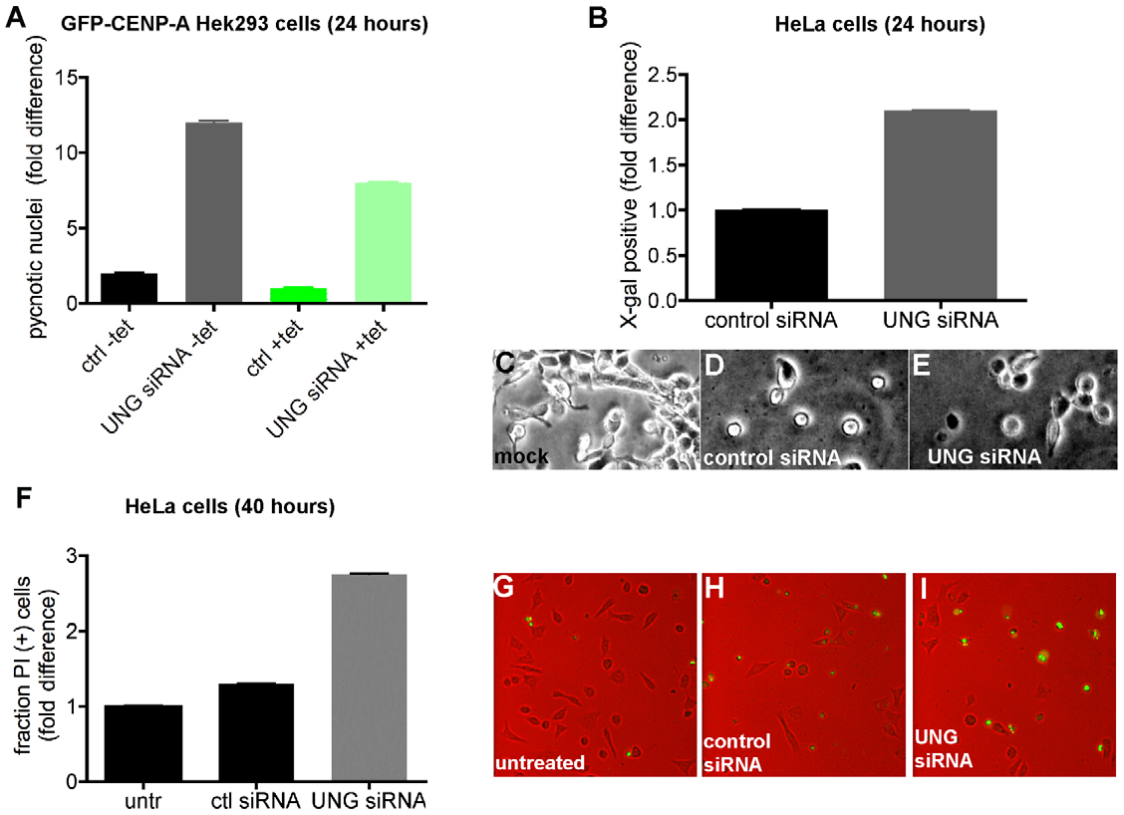
Depleting or inhibiting UNG resulted in apoptotic-like cell death after 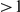 cell cycle. A. Manual quantitation (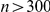 cells per sample) of pycnotic (hypercondensed) nuclei detected in GFP-CENP-A cells using DAPI staining 24 hours after transfection with control or UNG-directed siRNA. Control uninduced cells (−tet) behaved similarly to induced (+tet) cells. B. Manual quantitation (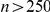 cells per sample) of X-gal staining detected in HeLa cells 24 hours after transfection with control or UNG-directed siRNA. Example images are shown in C–E. F. Manual quantitation (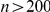 cells per sample) of propidium iodide (PI) staining of dead cells detected in HeLa populations 40 hours after transfection with control or UNG-directed siRNA. Example images are shown in G–H.

Although no significant cell cycle arrest was observed in cycling cells transfected with UNG2-directed siRNA (Figure 5C, top), the time-lapse imaging of H2B-YFP HeLa cells suggested that significant cell death was occurring when UNG2 depletion reached some critical threshold level. We reasoned that this should be detectable in the GFP-CENP-A inducible cell lines already characterized. Although the levels of cell death were not drastically elevated above the normal range for transfected cells, significant and reproducible differences in the fraction of hypercondensed or pycnotic nuclei were observed at 24 hours after transfection (Figure 6A). In multiple experiments, at 48 hours after transfection, all of the cells transfected with UNG2-directed siRNA were floating and apparently dead (SGZ, unpublished observations).

To further characterize the nature of this cell death, senescence-associated (SA) 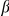-galactosidase activity was detected by X-gal staining in HeLa cells 24 hours after transfection with UNG2-directed siRNA. These experiments demonstrated that the cells transfected with UNG2-directed siRNA consistently displayed a 2-fold increase in X-gal positive staining compared to cells transfected with control siRNA (Figure 6B, example images shown in C–E). Here again, as seen in the Hek293 cells, at 48 hours after transfection, samples that received the UNG2-directed siRNA were floating and apparently dead (SGZ, unpublished observations). Since the association of SA-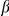-gal with apoptosis is somewhat controversial, propidium iodide exclusion experiments were performed at 40 hours after transfection. These results were consistent with the SA-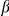 gal staining in that the fold difference of positively-stained cells was approximately 2 times higher in cells transfected with UNG2-directed siRNA than with controls (Figure 6F, examples shown in G–H). Taken together, these observations support the interpretation that depletion of UNG2 induces cell death.

### UNG2 transiently associates with centromeres during early G2 in human cells

**Figure 7 pone-0017151-g007:**
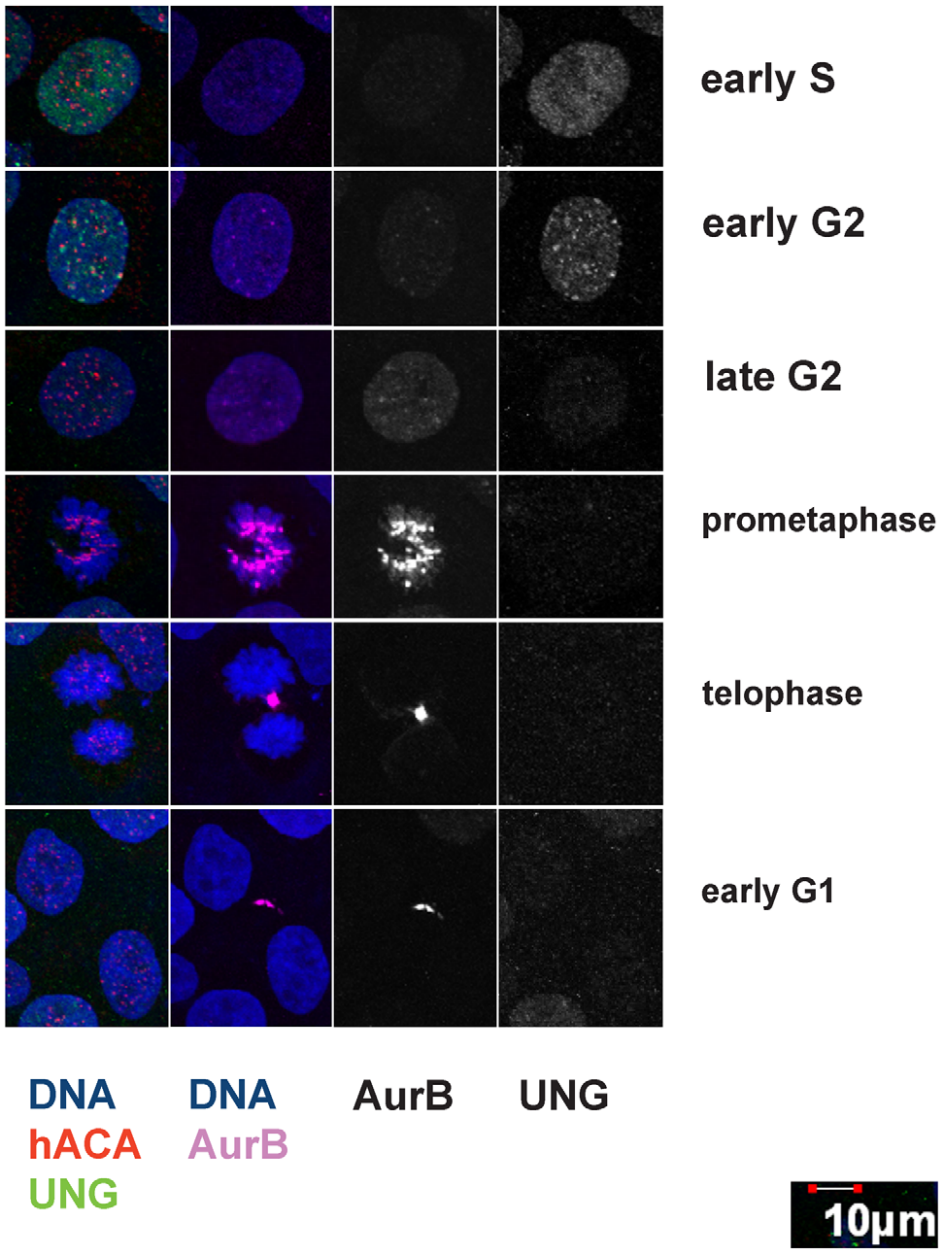
Cell cycle staging of UNG2 localization during G2 using indirect immunofluorescence. Example images from high-resolution confocal imaging using four channels. Left column, DNA was detected with DAPI (blue), endogenous CENP-A with ACA (red), and endogenous UNG2 with PU59 (green). Second column from the left, Aurora B was detected with Cy5 (pink) shown here with DNA (blue). Aurora B and UNG2 channels are shown alone in grayscale for clarity. Note the presence of Aurora B in the second row, indicating that UNG2 foci resembling centromeres are prominent during early G2.

It was previously reported that UNG2 accumulates in replication foci [43], and we detected UNG2 protein colocalized with centromeres in Xenopus egg extracts and cultured somatic cells [5]. Based on timing and morphological criteria, these observations suggested that UNG2 forms foci during late S phase or early G2. Association of UNG2 with centromeres during late S-phase would suggest a role for UNG2 during or immediately following replication of centromeric DNA. Alternatively, association of UNG2 with centromeres during G2 might coincide with the peak in CENP-A expression [44]. Since the kinase Aurora B was characterized in great detail as an early G2 marker [45], we used this protein and the nuclear localization of UNG2 to stage cells from S phase through early G1 (Figure 7). This analysis demonstrated for the first time that UNG2 focus formation coincides with the early stages of Aurora B localization at centromeres, during early G2. Consistent with other reports [35], [46], [47], UNG2 disappeared by late G2 and remained undetectable through early G1. In fact, the colocalization of UNG2 with centromeres was so striking that further analyses were performed to characterize this pattern in more detail.

### Transient UNG2 colocalization with centromeres correlates with the transition to double-dots

**Figure 8 pone-0017151-g008:**
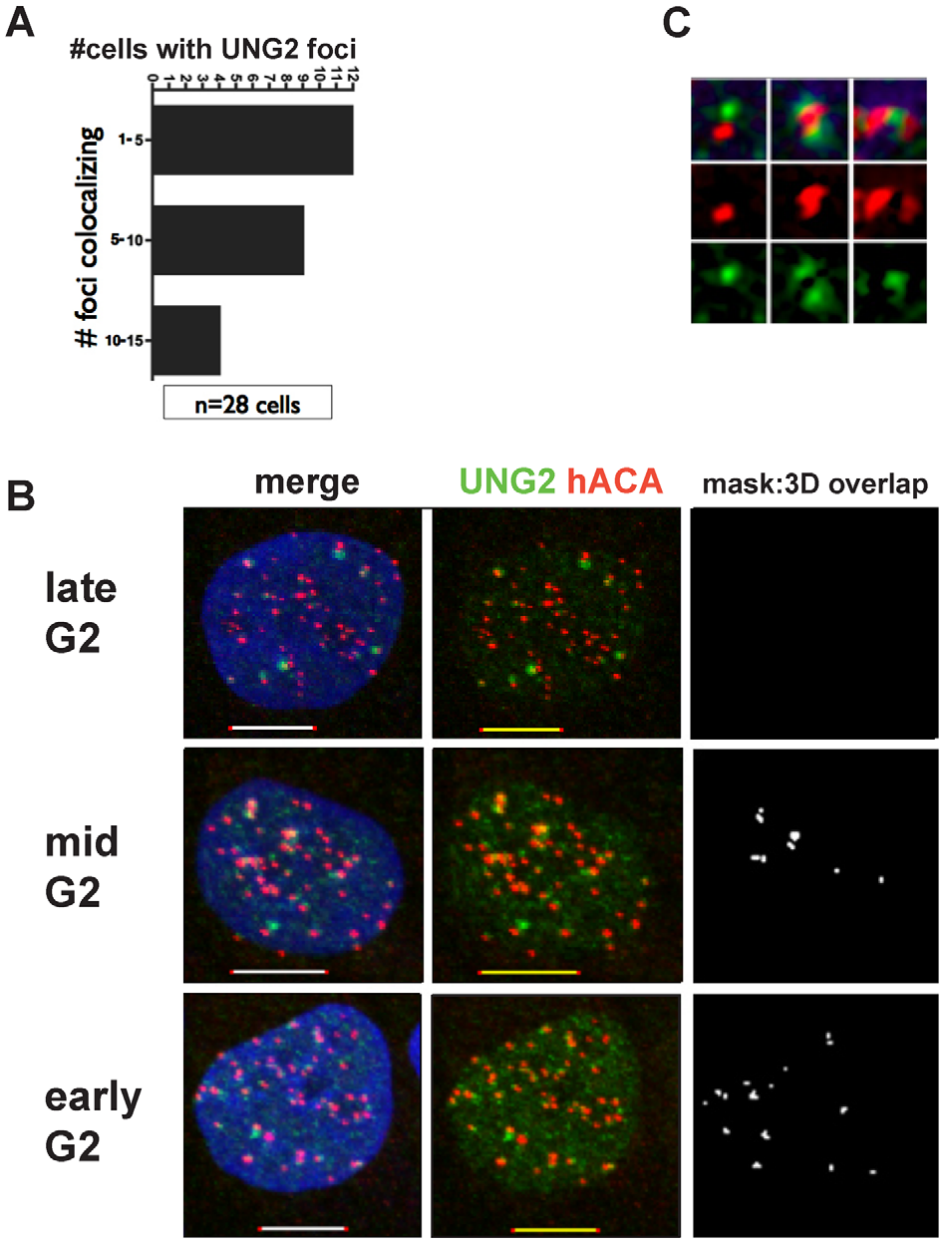
Transient UNG2 colocalization with centromeres correlates with the transition to double-dots. A. Binned categories of cells (n = 28 cells) with significant numbers of UNG2 foci colocalizing with centromeres. B. Example deconvolved high-resolution images of 143b cells in each bin. Colocalization of UNG2 detected with PU59 (green) with centromeres detected with ACA (red), shown with DNA (blue, left) or a mask of the measured 3-dimensional overlap above threshold (white, right). C. Examples of double-dots with UNG2 in green and ACA in red.

Since the fraction of cells exhibiting UNG2 colocalization with centromeres was very small (0.5% in a freely cycling cell population), this suggests that the duration of UNG2 dwell time at centromeres corresponds to approximately 5 minutes out of each 24-hour cell cycle. Furthermore, the number of foci colocalizing was variable. A series of 28 3-dimensional image stacks were quantitatively analyzed (see Methods) for the significance of colocalization as a defined amount of pixel overlap (Figure 8A). This analysis revealed that the majority of cells with significant colocalization displayed only 1–5 colocalizing foci, while slightly fewer cells exhibited 5–10 colocalizing foci, representing mid-G2, and only a minority exhibited 10–15 colocalizing foci, representing early G2 (Figure 8B). Interestingly, the appearance of UNG2 near centromeres also coincides with the transition from single to double-dots [48], long thought to be a hallmark of centromere protein assembly (Figure 8C).

### H2AX phosphorylation around centromeres in human cells

Our previous work suggested that the function of UNG2 in removing uracil from DNA was related to CENP-A assembly, at least in vitro [5]. More recently, DNA damage was shown to trigger CENP-A assembly in vivo [7]. These observations raised the possibility that UNG2 might participate in regulated, periodic DNA repair at centromeres to promote CENP-A assembly in every cell cycle, similar to its proposed roles in somatic hypermutation and class-switch recombination.

**Figure 9 pone-0017151-g009:**
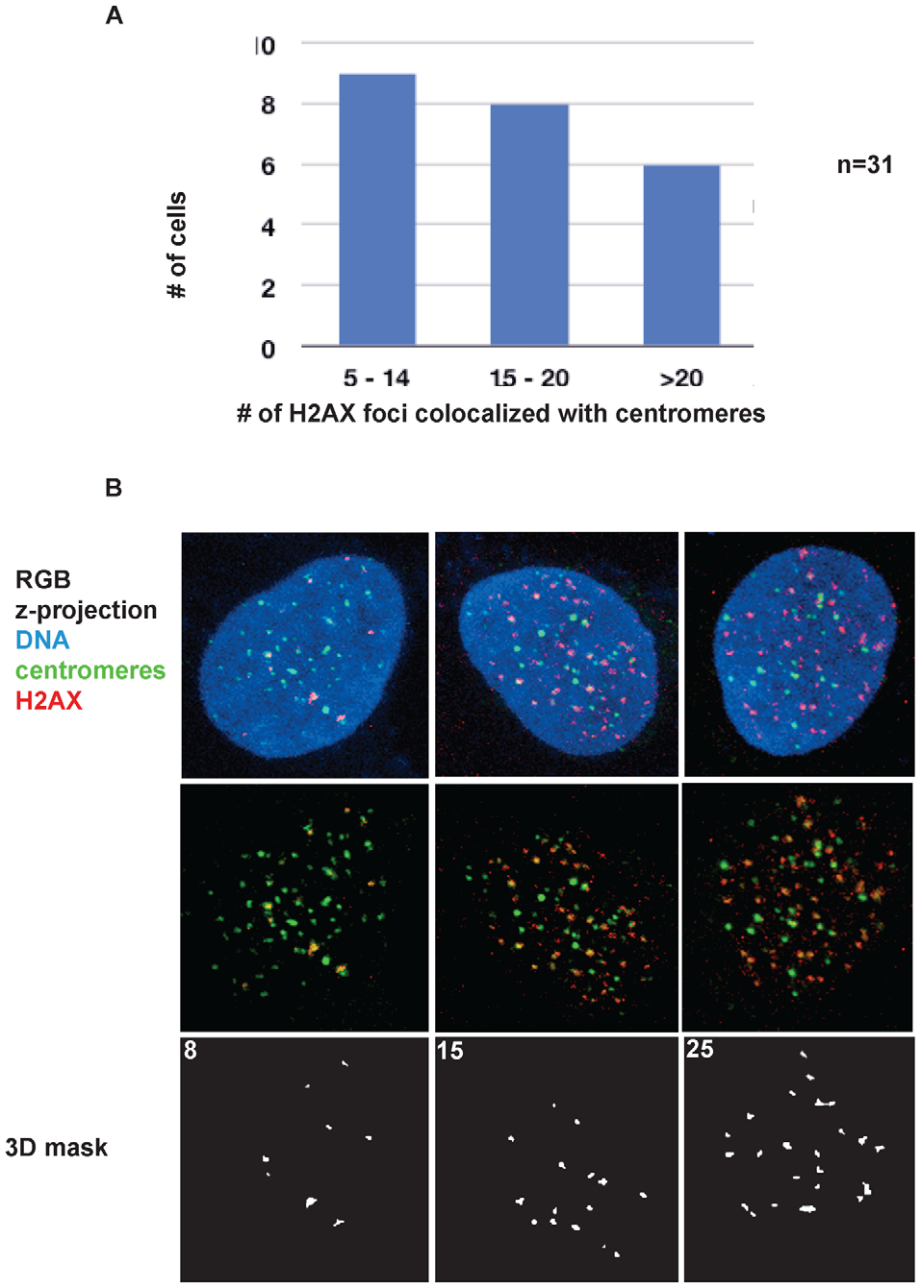
Quantitative analysis of phosphorylated H2AX signals colocalized with centromeres. A. Binned categories of cells (n = 31 cells) with significant numbers of phospho-H2AX foci colocalized with centromeres. B. Example deconvolved high-resolution images of 143b cells in each bin. Colocalization of centromeres detected with ACA (green), phospho-H2AX was detected with a monoclonal antibody (red), shown with DNA (blue, top row) or a mask of the measured 3-dimensional overlap above threshold (white, bottom row).

One prediction of this model would be that DNA damage signaling would be detectable at endogenous centromeres. To test this, H2AX phosphorylation was detected along with centromeres. This analysis revealed that out of 31 cells with visible colocalization, the largest proportion (9 cells) displayed H2AX phosphorylation signals colocalizing with 5–14 centromeres (Figure 9A). Slightly fewer (8 cells) displayed H2AX phosphorylation signals colocalizing with 15–20 centromeres, and a minority (6 cells) displayed H2AX phosphorylation signals colocalizing with 20 or more centromere foci (examples are shown in Figure 9B). These proportions, much higher than those observed for UNG2 colocalization with centromeres, are consistent with the observation that H2AX phosphorylation can persist for hours after DNA damage is incurred [49], much longer than the dwell times of DNA repair enzymes.

### Endogenous UNG2 and CENP-A colocalize with H2AX phosphorylation at centromeres and lines of laser-induced DNA damage

**Figure 10:**
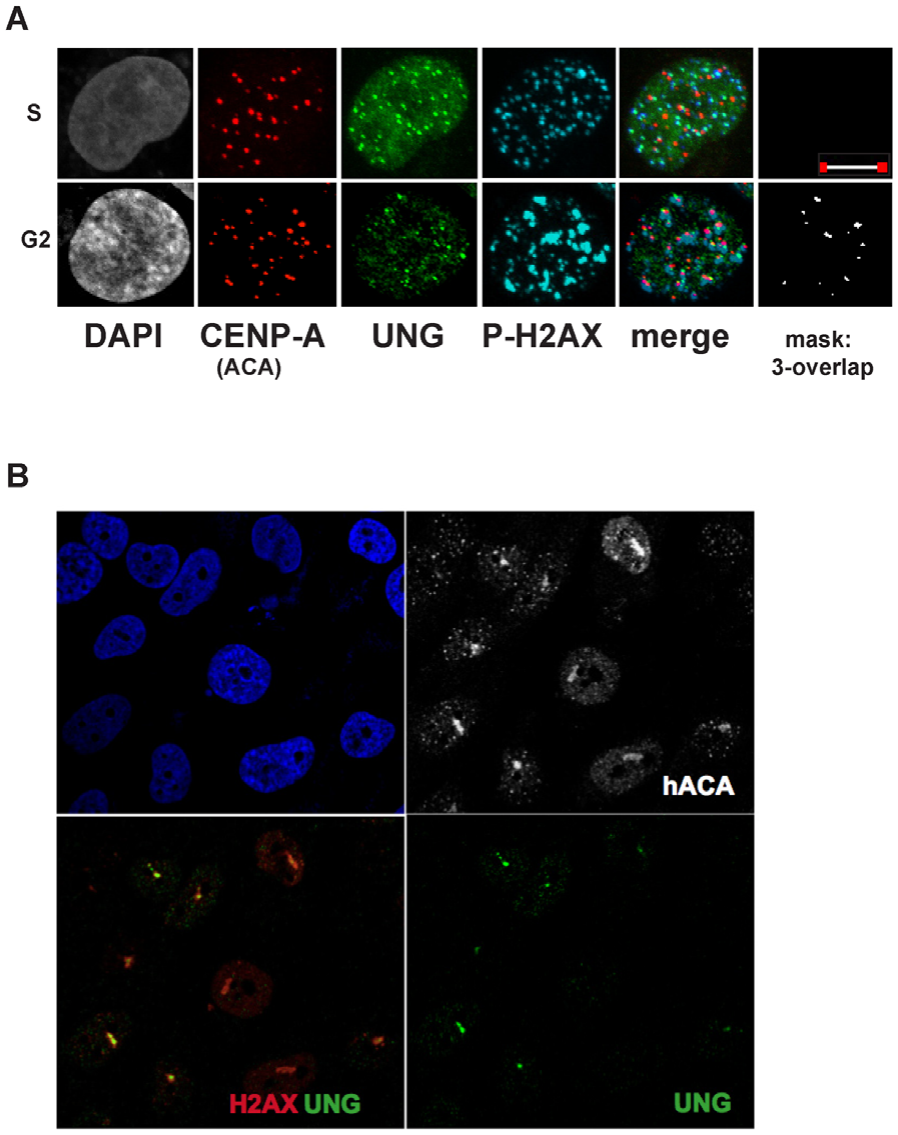
Endogenous UNG2 transiently colocalizes with CENP-A and phospho-H2AX at centromeres and lines of laser damage. A. Example deconvolved high-resolution images of 143b cells exhibiting different patterns of UNG2 localization. Top row images resemble S-phase replication foci with colocalization of UNG2 (green) with phospho-H2AX(blue) but not with CENP-A (red). Bottom row images demonstrate detectable three-way colocalization of CENP-A (red) with UNG2 (green) with phospho-H2AX (blue), as indicated by the measured overlap mask (white). Scalebar = 10 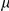
m B. Example confocal images of 143b cells subjected to laser exposure using methods described elsewhere [7]. Top left, nuclei detected with DAPI (blue). Bottom left, colocalization of phospho-H2AX (red) with UNG2 (green) in lines of laser damage. Top right, endogenous CENP-A (detected with ACA) is shown alone (white) to mark the lines of laser damage and centromeres (foci). Bottom right, UNG2 channel alone (green).

Since UNG2 is proposed to function in post-replicative repair and reported to colocalize with replication foci [50], we wondered whether the H2AX phosphorylation was occurring during S phase or G2. The characteristic pattern of numerous, bright UNG2 foci colocalized with H2AX phosphorylation indicates replication factories, along with pan-nuclear signal consistent with S-phase [50], however CENP-A did not colocalize with these structures in 143b cells (Figure 10A, top row). In contrast, the later stages of UNG2 patterns during early G2 (as shown in Figure 7) colocalized both with centromeres and with H2AX phosphorylation signals (Figure 10A, bottom row). These observations demonstrate that the presence of UNG2 at centromeres coincides with a marker of DNA damaging signaling and chromatin disruption. It is important to note, however, that H2AX does not appear to co-assemble with CENP-A [7].

Coincidence with H2AX phosphorylation suggested that UNG2 might be recruited to sites of laser-induced DNA damage along with CENP-A [7]. To examine this possibility, 143b cells were subjected to lines of laser-induced DNA damage (Figure 10B). This cells were then fixed with formaldehyde, and endogenous UNG2 (green), H2AX phosphorylation (red) and CENP-A (grayscale) were detected by indirect immunofluorescence, along with DNA (DAPI, shown in blue). Taken together, these experiments demonstrate UNG2 colocalization with CENP-A, both at centromeres and at sites of exogenous DNA damage.

### Overexpressing UNG2 is sufficient to rapidly induce high levels of GFP-CENP-A accumulation

If UNG2 is inducing DNA damage and/or chromatin disruption at centromeres, we reasoned that its overexpression might promote high levels of CENP-A assembly, similar to the increased number of foci observed in Xenopus extracts in response to DNA damage [5].

To perform this experiment in human cells, we co-transfected GFP-CENP-A and mCherry-tagged UNG2 into 143b osteosarcoma cells. [It is important to note that this experiment was unsuccessful in the GFP-CENP-A inducible cell lines and in some HeLa cell clones, apparently because the overexpression of UNG2 is toxic to many cell lines, SGZ unpublished observations.] In cells transfected only with GFP-CENP-A, the localization was consistent with both centromeres and some diffuse nuclear signal, as observed in other cell lines and previous reports [7].

**Figure 11:**
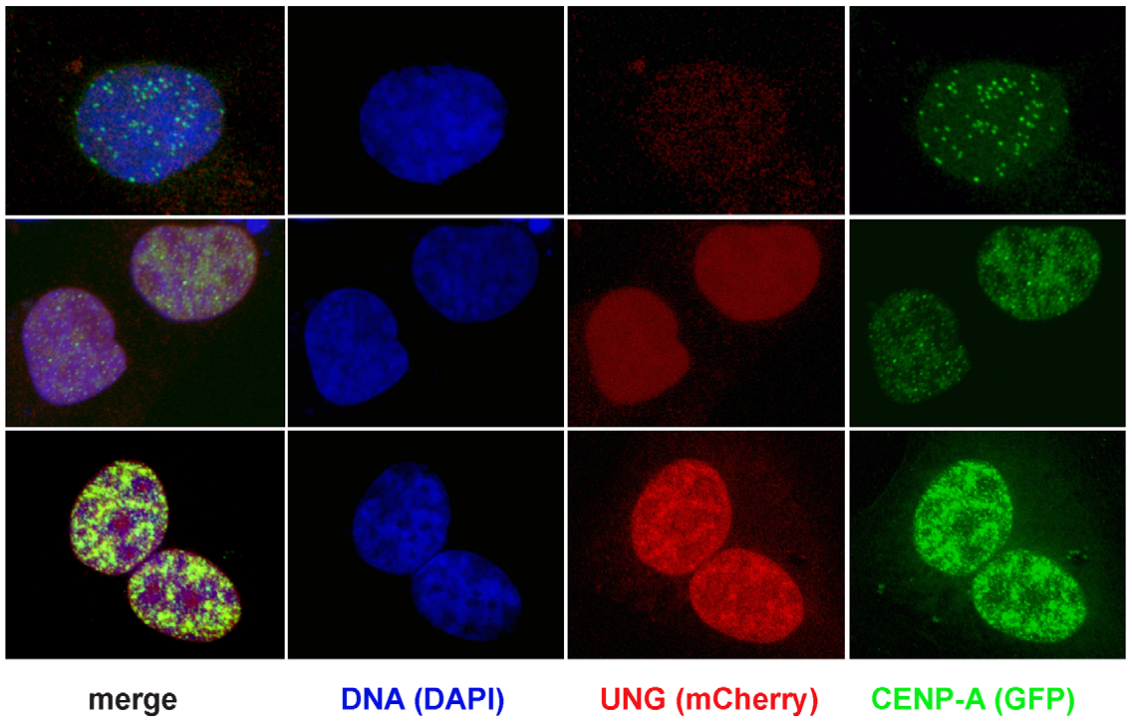
Overexpressing UNG-mCherry is sufficient to rapidly induce excess accumulation of GFP-CENP-A. Example images of 143b cells transiently co-transfected with mCherry-UNG (red) and GFP-CENP-A (green), fixed and stained with DAPI to detect DNA (blue). In cells with no detectable UNG, GFP-CENP-A localized to centromeres (top row). In cells with low levels of mCherry-UNG, GFP-CENP-A was distributed throughout the nucleus (middle row). In cells with high levels of mCherry-UNG, GFP-CENP-A accumulated to extremely high levels (bottom row).

In cells transfected with both GFP-CENP-A and mCherry-UNG2, several different patterns were observed, and these correlated with the level of mCherry-UNG2. Cells with low levels of mCherry-UNG2 exhibited similar patterns to cells transfected with GFP-CENP-A alone (Figure 11, top row). In contrast, cells with visible mCherry-UNG2 exhibited fewer foci and more generalized nuclear signal (Figure 11, middle row), while cells with high levels of mCherry-UNG2 exhibited unprecedentedly high levels of GFP-CENP-A accumulation (Figure 11, bottom row). These accumulations far surpassed the patterns seen from GFP-CENP-A overexpression alone, or MG132 treatment (compare with Figure 3 E and G). These observations are consistent with the hypothesis that UNG2 drives CENP-A assembly.

### Requirements for UNG2 accumulation at sites of DNA damage

Previously, we reported that the catalytic activity of recombinant UNG2 was required for CENP-A assembly in Xenopus egg extracts [5]. Since overexpressing mCherry-UNG2 was sufficient to induce very high levels of GFP-CENP-A accumulation in human 143b cells, we tested whether the catalytic activity was required for UNG2 accumulation at sites of DNA damage created using a multiphoton laser [7].

**Figure 12:**
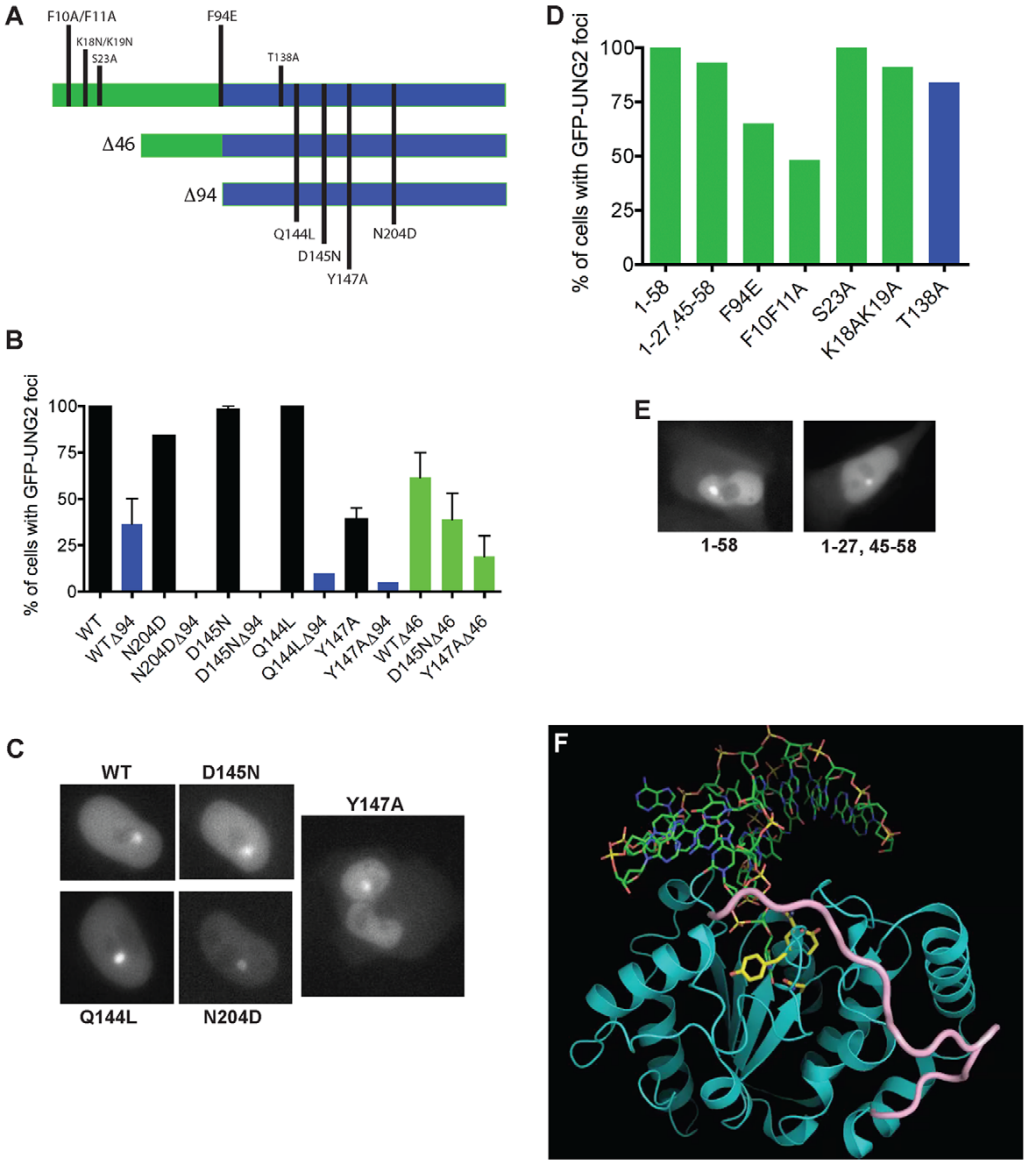
Requirements for UNG2 accumulation at sites of DNA damage. A. Schematic representation of truncations and point mutations tested in this study. B. Percent of cells with GFP-UNG2 foci at sites of laser-induced damage are shown, along with example images of the full-length catalytic site mutants. Full-length mutants (black bars) were all competent for accumulation, except for Y147A. 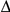
46 truncations (green bars) exhibited decreased focus formation. 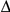
94 truncations (blue bars) were consistently the least competent, particularly the D145N and N204D versions. C. Example images of full-length mutants shown in part C. D. Percent of cells with GFP-fusion foci at sites of laser-induced damage, using N-terminal peptides lacking the catalytic domain (example images in part E), or non-catalytic point mutations in full-length UNG2. E. Example images of two mutants shown in part D. F. Molecular dynamics model showing that the N-terminal residues of UNG2 (residues 65–85; pink), could potentially interact with the catalytic domain (structures 1AKZ, 1EMH, 1SSP, [27], near active site residue Y147 (pink).

A series of previously generated mutants, based on the GFP-UNG2 construct [50], [51], were transfected into human 143b cells. The mutants tested included several active site mutations Q144L, D145N, Y147A, and N204D (Figure 12A). Note that the numbering used for these mutants is based on the mitochondrial UNG1, which includes a 9 residue N-terminal mitochondrial localization signal, which is subsequently cleaved off [50].

These mutants were subjected to multiphoton laser exposure under physiological conditions of heat and CO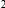, as described previously [7]. In these assay, wild-type full-length UNG2 exhibited rapid focus formation in 100% of the cells (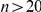 cells per experiment, 5 experiments). The average dwell-time was 20 minutes, after which some of the foci began to disappear.

Surprisingly, two of of the mutants, D145N and Q144L, which have 1000-fold lower catalytic activity than wild-type in vitro [26], rapidly formed foci in all of the cells exposed to laser-induced DNA damage (Figure 12B, 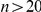 cells per experiment, 2 experiments each). The N204D mutant was slightly less efficient at focus formation (84% of cells, 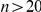 cells per experiment, 2 experiments), and the Y147A mutant was noticeably defective at focus formation (50% of cells, 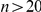 cells per experiment, 3 experiments). Other groups have previously proposed a role for Y147 (Y66 in E. coli UDG) in product release [51], [52], and it has been proposed that residues in the active site (D145 and N204) can interact with the N-terminus [31]. Based on these reports, we reasoned that interactions of the N-terminus with Y147 in the active site might affect targeting of UNG2 to chromatin.

To test whether Y147 might interact with the N-terminus of full-length UNG2 in vivo, we generated a series of truncation mutants, which were transfected into 143b cells and subjected to laser exposure. Although deletion of the N-terminus was not sufficient to completely inhibit focus formation by the wild-type catalytic domain, successive truncations did result in decreased focus formation (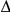
46 and 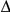
94, Figure 12B).

In the active site mutants, deletion of the N-terminus had a drastic effect not seen with the wild-type protein. For example, while D145
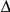
46 was approximately equivalent to the wild-type catalytic domain (WT
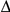
94) in focus formation frequency, the D145
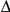
94 did not form foci at all (Figure 12B). Similar results were seen with the 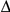
94 versions of Y147A, Q144L and N204D, which were all largely deficient in focus formation. These results suggest that the N-terminus of UNG2 provides an alternative mechanism for recruitment to chromatin when the active site is mutated.

These observations further raised the possibility that the N-terminus of UNG2, which has never been crystallized, might be capable of focus formation on its own. To test this, we used two constructs that were made for an earlier study on the nuclear import of UNG2 [50]. Both of these peptides, 1–58 and 1–27,45–58 formed foci at high efficiency in the laser assay (Figure 12C).

We also tested two mutants that disrupt known phosphorylation sites, S23A [47] and T138A (part of an S/T-Q sequence, a predicted PI3-like kinase substrate), but neither of these mutants were significantly impaired from focus formation.

From structure-gazing we hypothesized that F94 might be an important part of a hydrophobic pocket, especially if the N-terminus folds back over the active site. Mutation of this site to a charged residue (F94E) reduced the focus formation efficiency to 65% (n = 23 cells). Furthermore, the previously characterized PCNA/RPA binding site or so-called PIP box [53] includes F10 and F11 in the N-terminus. Mutation of these two residues to alanine (an established method of disrupting the PIP box, reviewed in [54] reduced focus formation efficiency to 48% (n = 21 cells). These experiments strongly suggest that the N-terminus of UNG2 is important for interactions with chromatin, not all of which are accounted for by interactions with PCNA or RPA.

## Discussion

Previously, we observed that inhibiting UNG2 in Xenopus egg extracts was sufficient to block assembly of CENP-A, a histone H3 variant [5]. At the time, UNG mutants were known to be viable in all species, and since no previous work had implicated DNA repair in CENP-A assembly, our initial report was met with skepticism. Since then, we directly tested this model and demonstrated that as little as one double-strand break can recruit CENP-A in living cells [7]. In addition, multiple other proteins have been implicated in CENP-A assembly, including at least one (HJURP) that was originally identified as having a function in DNA repair (reviewed in [6]). We have determined that HJURP is recruited to sites of DNA damage on a much slower timescale than UNG2 or CENP-A, suggesting that HJURP is not the primary CENP-A assembly factor (Figure S1). In contrast, the evidence presented here demonstrates that UNG2 recruitment to sites of DNA damage is extremely rapid and efficient.

There are two models for the role of UNG2 in CENP-A assembly, and the evidence presented here supports the possibility that the paradox can be reconciled by further studies. The first model is based on that of repetitive DNA at Ig loci (reviewed in [55], and proposes that uracil is generated in centromeric DNA by cytidine deamination. This model is supported by our evidence from Xenopus egg extracts, where CENP-A assembly was blocked not just by inhibiting UNG2, but also by zebularine [5]. In this model, uracil would be removed from DNA by UNG2, and removal of clustered uracils is known to be sufficient to generate double-strand breaks [33], [56]. CENP-A would then be recruited by binding directly to the damaged DNA, or via binding to other repair proteins [7].

In this model, CENP-A assembly would be expected to occur downstream of UNG2 action, such that CENP-A most likely would not bind directly to UNG2. Two observations are consistent with this. First, we have been unable to co-precipitate CENP-A directly bound to UNG2 (SGZ, unpublished). Second, the kinetics of the laser assay demonstrate that UNG2 forms foci faster than CENP-A, and CENP-A forms foci within minutes [7]. Interestingly, the proposed CENP-A “assembly” factor, HJURP [56], [57], seems to form foci around the time when UNG2 begins to disappear (approximately 20 minutes on average). This model would be consistent with the hypothesis that HJURP is required to stabilize CENP-A [6].

Alternatively, it is possible that UNG2 binds to double-strand breaks generated by other mechanism(s) [29], [30] via the N-terminal tail [31]. This model suggests that the presence of uracil is not required. Support for this model includes the multiphoton laser experiments shown here (Figures 11 and 12), as well as evidence in other model systems indicating that loss of UNG2 results in increased sensitivity to ionizing radiation [23], [58], which primarily produces double-strand breaks. In this model, UNG2 and CENP-A might co-exist transiently in repair foci, and UNG2 might be directly involved in CENP-A recruitment to damaged DNA or repair complexes.

The results presented here suggest that UNG2 catalytic activity may not be required for the localization of UNG2 to sites of DNA damage, and instead we propose that UNG2 has two modes of binding: via uracil in the active site, or via the N-terminal tail. Our earlier results in Xenopus egg extracts indicated that inhibition of UNG2 catalytic activity was sufficient to block CENP-A assembly (see Figures 5, 6 in [5]). The evidence presented here suggests that either of two binding modes are sufficient for UNG2 focus formation at sites of DNA damage.

## Methods

### Cell culture

Flp-In Hek293 parental cells (Invitrogen) were maintained in DMEM with 10% tetracycline-free serum (Hyclone, cat. no. SH30070) + penicillin/streptomycin. Hygromycin B(200 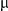
g/ml final concentration) was added for selection and maintenance of CENP-A-GFP clonal lines as described elsewhere [7]. HeLa and 143b (human osteosarcoma) cells were maintained in DMEM with 10% FBS, penicillin/streptomycin + nonessential amino acids (Gibco). All cell lines were tested for mycoplasma using the Plasmocin kit from InvivoGen.

### Reagents

Tetracycline (EMD Biosciences) was prepared as a 10 mg/ml stock in 70% sterile ethanol, protected from light exposure, and stored at 4

C for up to 1 month; MG132(EMD Biosciences) was prepared as a 1000×stock = 10 mM in DMSO, stored at −20

C. SiRNA (Ambion cat. no. 16708) against CENP-A (siRNA ID: 10613 and 10703), UNG (siRNA ID:139934) and matching Taqman detection kits (Applied Biosystems) were stored in small aliquots at −80

C and −20

C, respectively. Control Alexa Fluor 555 siRNA (Qiagen, cat. no. 1027099) was stored in small aliquots at −80

C.

### Indirect immunofluorescence

Antibodies: CENP-A (rabbit, affinity-purified polyclonal, 1∶100, cat. no. 07574, Upstate Biotech); human anti-centromere autoantibodies (1∶500–1∶1000, hACA, patient 83JD, a gift from Kevin Sullivan, Galway University); anti-HA mouse monoclonal antibody for detection of tagged Vpr constructs (Roche, clone 12CA5, cat. no. 1583816). Cells were fixed with 1% formaldehyde for 15 minutes or 4% formaldehyde for 10 minutes in PBS at room temperature. Immunofluorescence was performed as described elsewhere [7], [45], with DAPI and Slowfade mounting media from Molecular Probes, secondary antibodies from Jackson Immunoresearch.

### Microscopy and Image Presentation

Deconvolution microscopy (Figures 1 and 3) was performed on Deltavision microscopes in the UCSD Neuroscience Microscopy Shared Facility. Raw Deltavision 338 stacks of z-sections (.r3d) were deconvolved (15 iterations, conservative). Both raw (.r3d) and deconvolved (.r3d d3d) Deltavision files were displayed in SoftWorX Explorer (Applied Precision) for conversion and output as TIF files. TIF files were resized and figures were compiled using Adobe Photoshop and Adobe InDesign. Colocalization masks (Figures 8, 9, 10) were generated using ImageJ plugins. Confocal microscopy (Figures 1D–E, 4B, 6–10) was performed on an Olympus FV-1000. Automated microscopy (Figures 2,3,5) was performed on a Cellomics ArrayScan, essentially as described elsewhere [7]. For cell cycle analysis, cells transfected in Greiner 12-well culture dishes (cat. no. 665180) were fixed and stained with DAPI before imaging with a 20× Zeiss lens and an ORCA-ER CCD camera. Cells were identified using the DAPI channel and the Target Activation module (Cellomics HCS software). Data were exported as Excel spread sheets and raw .tif files for further analysis (see Statistical Methods).

### Western detection

SDS-PAGE was performed using Novex precast gels from Invitrogen (4–20% tris-glycine gradient gels). Gels were transferred to PVDF (Biotrace, Pall) in TowbinÕs transfer buffer in a Novex transfer apparatus at 35 V for 4 hours at room temperature. Transfer was confirmed with Ponceau S stain (Sigma). PVDF membranes were incubated with antibodies as described previously [45]. Antibodies were incubated with membranes for 1 hour at room temperature, unless stated otherwise. Anti-GFP mouse monoclonal (1∶1000, Roche), Anti-CENP-A affinity-purified rabbit polyclonal peptide antibody (1∶1000, Upstate cat. no. 07574, incubated at 4

C overnight), anti-H3 C-terminal epitope rabbit polyclonal (1∶1000, Upstate cat. no. 07690). Anti-tubulin mouse monoclonal (1∶1000–1∶2000, clone Dm1a mouse monoclonal ascites fluid, Sigma cat. no. T9026), Anti-UNG (1∶2000, PU59 affinity-purified rabbit polyclonal, [24]).

### Transfections, RNA preparation and gene expression analysis

Transfections were performed in suspension using Lipofectamine2000 (Invitrogen). For a 24-well dish, 200,000 cells were transfected with 20 pmol of siRNA or 200 ng of plasmid DNA and 0.5–1 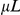 of Lipofectamine2000 in 1 ml Optimem (Invitrogen). Cells were plated on No. 1.5, 10 mm glass coverslips (Fisher) that were acid and ethanol-washed, or glass-bottom dishes (Nunc). In all cases, cell substrates were coated with fibronectin, as previously described [7]. Transfection efficiency was verified by counting cells positive for Alexa Fluor 555 labeled siRNA or by GFP/YFP-tagged protein, using DAPI as a counterstain, on a Zeiss Axioskop.

RNA samples were prepared and gene expression analyzed as described previously [7].

### Statistical analyses

For all figures, error bars represent standard deviations, unless otherwise stated. P-values were calculated using the unpaired t-test (Figure 4C, 5F) when comparing two independent samples, or the one-way ANOVA (Figure 5E) when comparing more than two samples, with Graphpad Prism software.

Probability Binning analysis of Cellomics image intensities (Figure 2E) was performed according to published methods [59]. The probability binning test (PB) is a variant of chi-squared that yields a T value, which is used to rank distributions by similarity to a control sample. Briefly, a T value of 0 indicates that two distributions are identical, a value of 1 indicates that two distributions are within one standard deviation of each other, while higher values indicate that two distributions are significantly different. Bins were selected to contain equal numbers of cells, where each bin represents 10% of the intensity range for all the control (mock-transfected) cells. These bins were then used to analyze the other two samples (control siRNA and UNG siRNA). Using the control siRNA or using all the cells in all samples for creating bins yielded similar results (not shown). T values were calculated according to published methods [59], using a custom Ruby script as well as the open-source statistics package R, which also provided p-values. Both methods yielded the same results. Graphs shown in Figure 2E were generated using GNU Plot.

### Assays for cell death

HeLa cells were transfected in suspension and plated on a fibronectin-coated 12-well plastic dish at low cell density (100,000 cells per well). Samples prepared in this way were subjected to either senescence-associated 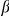-galactosidase staining or propidium iodide exclusion, followed by imaging and manual cell counting.

Senescence associated 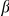-galactosidase staining was performed consistent with published protocols [60]. Briefly, cells were fixed 5 minutes at room temperature in PBS with 4% formaldehyde, then washed with PBS twice before incubation in staining solution overnight at 37

C. Staining solution: 30 mM citric acid/phosphate buffer, 5 mM 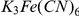, 5 mM 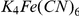, 150 mM 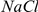, 1 mg/ml X-gal, 2 mM 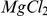). Citric acid/phosphate buffer: 0.1 M citric acid, 0.2 M sodium phosphate dibasic, pH 6.0. X-gal stock solution: 20 mg/ml in dimethylformamide, stored at −20

C.

40 hours after siRNA transfection, culture media was replaced with propidium iodide solution for 5 minutes in PBS. Only dead/dying cells take up propidium iodide without chemical permeabilization Note that these numbers are probably an underestimation since dead cells tend to float, and some are lost when the culture media is removed. Imaging was performed using the Cellomics ArrayScan with a 20× lens, 5 fields per well, each sample in triplicate. For Figure 6F, cells were counted manually using the ImageJ Particle Analysis Cell Counter plugin.

### Uracil-removing activity assays

500,000–600,000 cells were plated per well in 12-well dishes and transfected with 40 pmol siRNA (Ambion) and Lipofectamine2000 in Optimem (Invitrogen), according to the manufacturerÕs instructions. 24 hours later, cells were trypsinized and pellets were flash-frozen and stored at −80 C. Pellets were resuspended in 500 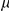 l homogenization buffer (10 mM Tris-HCl pH 7.5, 60 mM NaCl, 1 mM EDTA, 1 mM DTT, +complete protease inhibitors from Roche). Cell suspension was sonicated for 1 minute on ice (Branson mini-tip, 20% duty cycle, 2.5 output). Protein concentration of lysates was measured with the QuantIt protein assay (Invitrogen). Uracil removal was assayed as described previously [9] in buffer supplemented with 0.5 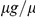 l BSA final, and 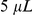 labeled DNA substrate.

### Molecular dynamics modeling

Modeller [61] was used to align structures of human UNG (consisting of residues 95–314), and to perform molecular dynamics simulations in order to calculate plausible locations of 20 additional residues (75-IQRNKAAALLRLAARNVPVG-94) from the N-terminus of human UNG. The model of the core catalytic domain is based alignments of structures 1AKZ (wild-type UNG2 residues 95–314), 1EMH (substrate complex) and 1SSP (product & product+U) [27]. To determine whether the N-terminal residues could interact with the catalytic domain, we refined the positions of residues 75–94, with the restraint that residue 75 be within 10 angstroms (CA-CA distance) of residue 147, using the loop refinement procedure in Modeller [62]. Approximately 33% of the resulting models had acceptable stereochemical and steric profiles, given the above restraints. Figure 12F shows a snapshot of the lowest energy configuration, representing the best fit model.

## Supporting Information

Figure S1 GFP-tagged HJURP was transfected into human 143b cells and subjected to laser exposure. The example cell shown here formed a focus at the site of laser exposure within 10 minutes. 30% of cells (n = 21 cells) formed foci, with an average time of 20 minutes (+/−11 minutes, std. dev). For methods, see [7].
(TIFF)

Movie S1 HeLa cells with integrated H2B-YFP were transfected with UNG-directed siRNA and subjected to live cell imaging overnight. Most cells did not survive more than one cell division, although cell death occurred at different times after nuclei were observed to separate.
(MOV)

Movie S2 HeLa cells with integrated H2B-YFP were transfected with UNG-directed siRNA and subjected to live cell imaging overnight. Most cells did not survive more than one cell division, although cell death occurred at different times. Some cells arrested in mitosis for various lengths of time, and then died.
(MOV)

Movie S3 HeLa cells with integrated H2B-YFP were transfected with UNG-directed siRNA and subjected to live cell imaging overnight. Mitotic defects were common.
(MOV)

Movie S4 HeLa cells with integrated H2B-YFP were transfected with UNG-directed siRNA and subjected to live cell imaging overnight. Some cells failed to execute a normal mitosis, resulting in nuclei of varying sizes.
(MOV)
