# Supplementary figures and images for: Uracil DNA N-Glycosylase Promotes Assembly of Human Centromere Protein A
Source: PLoS One. 2011 Mar 2;6(3):e17151. doi: 10.1371/journal.pone.0017151 (PMC3047565; doi:10.1371/journal.pone.0017151)

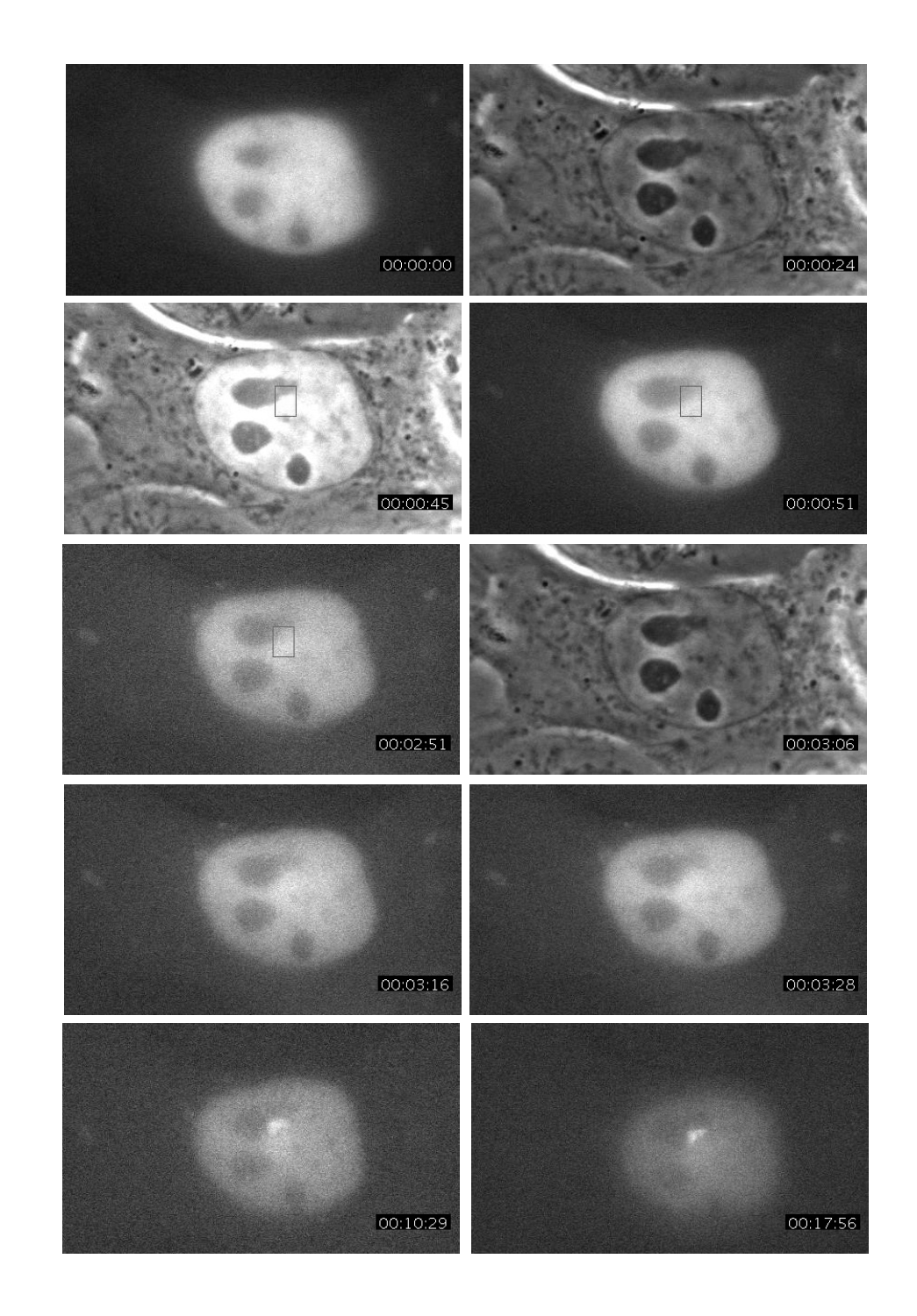

Supplement: Figure S1 — GFP-tagged HJURP was transfected into human 143b cells and subjected to laser exposure. The example cell shown here formed a focus at the site of laser exposure within 10 minutes. 30% of cells (n = 21 cells) formed foci, with an average time of 20 minutes (+/−11 minutes, std. dev). For methods, see [7]. (TIFF) [file pone.0017151.s001.tif]
